# Phenotypic Variation and Pubertal Outcomes in Males and Females With 46,XY Partial Gonadal Dysgenesis

**DOI:** 10.1210/clinem/dgaf223

**Published:** 2025-04-10

**Authors:** Rieko Tadokoro-Cuccaro, Ieuan A Hughes, Martine Cools, Koen van de Vijver, Berenice Bilharinho de Mendonça, Sorahia Domenice, Rafael Loch Batista, Renata Thomazini Dallago, Elaine F Costa, Nathalia Lisboa Gomes, Andréa T Maciel-Guerra, Gil Guerra-Junior, Juliana Gabriel Ribeiro de Andrade, Angela Lucas-Herald, Jillian Bryce, Sabine Hannema, Anders Juul, Evgenia Globa, Ken McElreavey, Federico Baronio, Rodolfo Rey, Jimena Lopez Dacal, Feyza Darendeliler, Sukran Poyrazoglu, Zofia Kolesińska, Marek Niedziela, Hedi L Claahsen–van der Grinten, Erica L T van den Akker, Gloria Herrmann, Navoda Atapattu, Vandana Jain, Rajni Sharma, Markus Bettendorf, Daniel Konrad, Nina Lenherr-Taube, Paul Martin Holterhus, Simona Fica, Mars Skae, Gianni Russo, Marianna Rita Stancampiano, Gabriella Gazdagh, Justin H Davies, Zainaba Mohamed, Sumudu Nimali Seneviratne, Tülay Güran, Ayla Güven, Malgorzata Wasniewska, Vilhelm Mladenov, Gilvydas Verkauskas, Renata Markosyan, Marta Korbonits, Olaf Hiort, Isabel Viola Frielitz-Wagner, S Faisal Ahmed, Ajay Thankamony

**Affiliations:** Department of Paediatrics, University of Cambridge, Cambridge, CB2 0QQ, UK; Department of Paediatrics, University of Cambridge, Cambridge, CB2 0QQ, UK; Department of Internal Medicine and Paediatrics, Ghent University, 9000 Ghent, Belgium; Department of Pediatric Endocrinology, Ghent University Hospital, 9000 Ghent, Belgium; Department of Pathology, Ghent University, 9000 Ghent, Belgium; Endocrinology Division, Internal Medicine Department, Development Endocrinology Unit, Hormone and Molecular Genetics Laboratory (LIM/42) and SELA (Laboratório de Sequenciamento em Larga Escala), Medical School, University of São Paulo, 05403-900 Sao Paulo, Brazil; Endocrinology Division, Internal Medicine Department, Development Endocrinology Unit, Hormone and Molecular Genetics Laboratory (LIM/42) and SELA (Laboratório de Sequenciamento em Larga Escala), Medical School, University of São Paulo, 05403-900 Sao Paulo, Brazil; Endocrinology Division, Internal Medicine Department, Development Endocrinology Unit, Hormone and Molecular Genetics Laboratory (LIM/42) and SELA (Laboratório de Sequenciamento em Larga Escala), Medical School, University of São Paulo, 05403-900 Sao Paulo, Brazil; Endocrinology Division, Internal Medicine Department, Development Endocrinology Unit, Hormone and Molecular Genetics Laboratory (LIM/42) and SELA (Laboratório de Sequenciamento em Larga Escala), Medical School, University of São Paulo, 05403-900 Sao Paulo, Brazil; Endocrinology Division, Internal Medicine Department, Development Endocrinology Unit, Hormone and Molecular Genetics Laboratory (LIM/42) and SELA (Laboratório de Sequenciamento em Larga Escala), Medical School, University of São Paulo, 05403-900 Sao Paulo, Brazil; Division of Endocrinology, Santa Casa de Belo Horizonte, Belo Horizonte 30150-221, Brazil; Department of Internal Medicine, Federal University of Minas Gerais, Medical School, Belo Horizonte 30130-100, Brazil; School of Medical Sciences, Interdisciplinary Group for Study of Sex Determination and Differentiation (GIEDDS), State University of Campinas (Unicamp), 13083-887 Campinas, Brazil; School of Medical Sciences, Interdisciplinary Group for Study of Sex Determination and Differentiation (GIEDDS), State University of Campinas (Unicamp), 13083-887 Campinas, Brazil; School of Medical Sciences, Interdisciplinary Group for Study of Sex Determination and Differentiation (GIEDDS), State University of Campinas (Unicamp), 13083-887 Campinas, Brazil; Developmental Endocrinology Research Group, University of Glasgow, Glasgow, G51 4TF, UK; Office for Rare Conditions, University of Glasgow, Glasgow, G51 4TF, UK; Department of Paediatric Endocrinology, Amsterdam UMC location Vrije Universiteit Amsterdam, 1081 HV Amsterdam, the Netherlands; Amsterdam Reproduction & Development, 1105 AZ Amsterdam, the Netherlands; Amsterdam Gastroenterology Endocrinology Metabolism, 1081 HZ Amsterdam, the Netherlands; Department of Growth and Reproduction, Copenhagen University Hospital-Rigshospitalet, 2100 Copenhagen, Denmark; Ukrainian Scientific and Practical Center of Endocrine Surgery, Transplantation of Endocrine Organs and Tissues of MOH of Ukraine, Kyiv 01021, Ukraine; Institut Pasteur, Université Paris Cité, Human Developmental Genetics Unit, Paris F-75015, France; Centre National de la Recherche Scientifique, CNRS, UMR 3738, Paris 75794, France; Department Hospital of Woman and Child, Pediatric Unit, Center for Rare Endocrine Conditions (Endo-ERN), IRCCS-Azienda Ospedaliero-Universitaria di Bologna, Bologna 40138, Italy; CONICET–FEI–Division of Endocrinology, Centro de Investigaciones Endocrinológicas “Dr. César Bergadá” (CEDIE), Hospital de Niños Ricardo Gutiérrez, C1425EFD Buenos Aires, Argentina; CONICET–FEI–Division of Endocrinology, Centro de Investigaciones Endocrinológicas “Dr. César Bergadá” (CEDIE), Hospital de Niños Ricardo Gutiérrez, C1425EFD Buenos Aires, Argentina; Department of Pediatric Endocrinology, Istanbul University Istanbul Faculty of Medicine, Istanbul 34098, Turkey; Department of Pediatric Endocrinology, Istanbul University Istanbul Faculty of Medicine, Istanbul 34098, Turkey; Department of Pediatric Endocrinology and Rheumatology, Karol Jonscher Clinical Hospital, Poznan University of Medical Sciences, 60-572 Poznan, Poland; Department of Pediatric Endocrinology and Rheumatology, Karol Jonscher Clinical Hospital, Poznan University of Medical Sciences, 60-572 Poznan, Poland; Department of Pediatrics, Radboud University Medical Centre, Amalia Children's Hospital, 6525 GA Nijmegen, The Netherlands; Department of Pediatrics, Division of pediatric endocrinology and DSD Center of expertise, Erasmus MC-Sophia Children’s Hospital, University Medical Center Rotterdam, 3015 CN Rotterdam, The Netherlands; Division of Pediatric Endocrinology and Diabetes, Department of Pediatrics and Adolescent Medicine, University Medical Center Ulm, 89075 Ulm, Germany; Department of Pediatrics, Lady Ridgeway Hospital, Colombo 08, Sri Lanka; Department of Pediatrics, Division of Pediatric Endocrinology, All India Institute of Medical Sciences, New Delhi 110029, India; Department of Pediatrics, Division of Pediatric Endocrinology, All India Institute of Medical Sciences, New Delhi 110029, India; Paediatric Endocrinology and Diabetes, Heidelberg University Children´s Hospital, 69120 Heidelberg, Germany; Division of Endocrinology and Diabetes, University Children's Hospital Zurich, University Zurich, 8032 Zurich, Switzerland; Division of Endocrinology and Diabetes, University Children's Hospital Zurich, University Zurich, 8032 Zurich, Switzerland; Department of Paediatric Oncology and Rheumatology, Paediatric Endocrinology and Diabetes, University of Schleswig-Holstein, Campus Kiel, 24105 Kiel, Germany; Endocrinology and Diabetes, “Carol Davila” University of Medicine and Pharmacy, Bucharest 050474, Romania; Department of Paediatric Endocrinology, Royal Manchester Children's Hospital, Manchester M13 9WL, UK; Department of Pediatrics, IRCCS San Raffaele Scientific Institute, 20132 Milan, Italy; Department of Pediatrics, IRCCS San Raffaele Scientific Institute, 20132 Milan, Italy; Wessex Clinical Genetics Service, Princess Anne Hospital, University Hospital Southampton NHS Trust, Southampton, SO16 5YA, UK; Department of Paediatric Endocrinology, University Hospital Southampton NHS Foundation Trust, Southampton, SO16 6YD, UK; Department of Paediatric Endocrinology and Diabetes, Birmingham Women's and Children's Hospital NHS Trust, Birmingham, B4 6NH, UK; Department of Paediatrics, University of Colombo, Colombo 08, Sri Lanka; Department of Paediatric Endocrinology and Diabetes, Marmara University, School of Medicine, 34841 Istanbul, Turkey; Emeritus Pediatric Endocrinologist, Başkent University Faculty of Medicine, Department of Child Health and Diseases, Istanbul Hospital, 34770 İstanbul, Turkey; Department of Human Pathology of Adulthood and Childhood, University of Messina, 98122 Messina, Italy; Department of Paediatrics - UMHAT ‘Sv.Marina’, Medical University of Varna, 9010 Varna, Bulgaria; Faculty of Medicine, Institute of Clinical Medicine, Vilnius University, 03101 Vilnius, Lithuania; Department of Endocrinology, Yerevan State Medical University, Yerevan 0025, Armenia; Endocrinology, Barts and The London School of Medicine and Dentistry, Queen Mary University of London, London, EC1A 7BE, UK; Division of Pediatric Endocrinology and Diabetes, Department of Pediatric and Adolescent Medicine, University of Lübeck, 23562 Lübeck, Germany; Division of Pediatric Endocrinology and Diabetes, Department of Pediatric and Adolescent Medicine, University of Lübeck, 23562 Lübeck, Germany; Developmental Endocrinology Research Group, University of Glasgow, Glasgow, G51 4TF, UK; Office for Rare Conditions, University of Glasgow, Glasgow, G51 4TF, UK; Department of Paediatrics, Addenbrooke's Hospital, Cambridge University Hospitals NHS Foundation Trust, Cambridge, CB2 0QQ, UK

**Keywords:** 46,XY gonadal dysgenesis, differences/disorders of sex development, virilization, gonadectomy, spontaneous puberty, sex reassignment

## Abstract

**Background:**

46,XY gonadal dysgenesis is classified as complete (CGD) or partial (PGD) subtypes. The phenotype of PGD and the long-term outcome is not clearly defined.

**Objective:**

To evaluate clinical features and pubertal outcome of PGD in a large cohort, using CGD as a comparator for diagnostic clarity.

**Methods:**

Patients with 46,XY GD were identified from the I-DSD Registry and data on phenotype, genetics, biochemistry, gonadal histology, and pubertal development were collated in 3 categories; CGD (n = 100), PGD assigned female (PGDf, n = 107), and male (PGDm, n = 103) at birth.

**Results:**

Most individuals with PGD presented with atypical genitalia in infancy, though, 18% of PGDf presented with delayed puberty and 8% with virilization. A genetic etiology was identified in 42% of the cohort, with common gene defects in *SRY* and *WT1* in CGD and *NR5A1* in PGD. Gonadal pre-/malignancy was found in 33.8% in CGD, 19.7% in PGDf, and 8.8% in PGDm. Among the PGDm (>13 years) with at least 1 gonad, 80% had spontaneous pubertal onset and 59% achieved Tanner G5 without hormone treatment. Labioscrotal gonads at presentation and testosterone response to human chorionic gonadotropin predicted onset of spontaneous puberty. In PGDf with gonads, 42% developed spontaneous virilization at puberty. Sex was reassigned in 16.1% and 5.3% of individuals with PGDf and PGDm, respectively.

**Conclusion:**

This study highlights the heterogeneous phenotype of PGD and the consequent diagnostic challenge. Many PGD patients with preserved gonads have the potential to develop puberty spontaneously, though further study is needed to determine the risk of developing gonadal tumors.

Gonadal dysgenesis (GD) is a term used when development of the gonad into a testis or an ovary is incomplete or impaired. It is a heterogeneous condition often associated with sex chromosomal anomalies or alterations in various genes involved in the development of the gonad (1). GD is classified as complete (CGD), partial (PGD), and gonadal regression depending on morphology and function (1). 46,XY GD is a common form of GD, in which testis differentiation and development has started but is incomplete.

In contrast to the well-characterized presentation of 46,XY CGD with typical female external genitalia and presence of Mullerian structures, the diagnosis of 46,XY PGD is challenging as the phenotype can overlap with other conditions under the umbrella of differences of sex development (DSD) (2). Diagnosis of PGD is based on a combination of clinical features, endocrine investigations, genetic studies, and gonadal histology where necessary (3). However, a recent study using the next-generation sequencing approach for known or candidate genes, found alterations in only 40% of partially virilized patients with 46,XY DSD (4). Furthermore, biopsy of the gonads is not always desirable, especially in individuals reared male.

Due to the rarity and heterogeneous presentation of 46,XY PGD, outcome data are limited. Two small studies have reported a high proportion of males (60%-80%) who attained spontaneous onset of puberty (5, 6). However, data on further pubertal development, gonadal function, and gonadal malignancies are limited.

We explored a large international dataset of patients to characterize clinical features of PGD using CGD as a comparator where a firm diagnosis can be made. Other aims of the study were to determine outcome at puberty, including gonadal histology and function and the frequency of sex reassignment.

## Patients and Methods

### Data Collection

Data were collected from the I-DSD registry and 2 additional centers. The I-DSD Registry is an international database established in 2011 and hosted at the University of Glasgow (https://sdmregistries.org/). It has >6671 cases from 120 centers from 43 countries (June 2022) and the research activity has recently been described (7). In brief, informed consent is obtained prior to enrollment and pseudonymized data from routine clinical care are stored in the registry (7). Centers with cases of 46,XY GD (n = 257) were contacted by e-mail between July 2021 and August 2021, with 2 reminders sent to centers that did not reply. A spreadsheet was used to collect information, containing 98 items for female gender and 121 items for male gender. Participating clinicians returned the completed files in a secured shared folder. The 2 additional centers were Hospital de Clínicas, State University of Campinas, Brazil, and Hospital das Clinicas, University of São Paulo, Brazil.

### Patients

We used case definitions as previously reported (8). Inclusion criteria for CGD were: (1) 46,XY karyotype, (2) typical female genitalia, (3) presence of Mullerian duct derivatives or undetectable anti-Mullerian hormone (AMH), and (4) if at pubertal age, elevated levels of LH/FSH (Table 1). Inclusion criteria for PGD were: (1) 46,XY karyotype, (2) atypical genitalia, and (3) at least 1 of the following features that suggests GD: (A) testosterone levels after human chorionic gonadotropin (hCG) stimulation test less than twice the basal value, low basal testosterone with high gonadotropin levels in minipuberty or puberty, AMH or inhibin B levels below male reference range, or presence of Mullerian duct derivatives; (B) histology consistent with GD; or (C) pathogenic variants in genes associated with GD (8) (Table 1). We assigned patients into 3 categories: CGD, PGD assigned female (PGDf), and PGD assigned male (PGDm) at birth.

**Table 1.** Inclusion criteria for CGD and PGD

	CGD: 1, 2, and either 3 or 4		PGD: 1, 2, and either 3 or 4, 5 or 6
1. Karyotype	46,XY	1. Karyotype	46,XY
2. External genitalia	Female type	2. External genitalia	Female type with no Mullerian duct derivatives Female type + virilization at puberty Atypical genitalia
3. Internal genitalia	Mullerian duct derivatives	3. Internal genitalia	Mullerian duct derivatives
4. Biochemistry	Undetectable anti-Mullerian hormone (AMH)	4. Biochemistry	One of the following; Testosterone level < twice the basal value after hCG Low basal testosterone with high gonadotropin levels in minipuberty or puberty AMH or inhibin B levels below male reference range
	Additional: elevated LH/FSH if pubertal age		
		5. Histology of gonad	Histology consistent with GD
		6. Genetic study	Pathogenic variants in genes associated with GD

Abbreviations: AMH, anti-Mullerian hormone; CGD, complete gonadal dysgenesis; GD, gonadal dysgenesis; hCG, human chorionic gonadotropin; PGD, partial gonadal dysgenesis.

Data were received on 221 patients from 34 centers through the registry and a further 113 patients from the 2 institutions (Campinas n = 48, São Paulo n = 65). We excluded 24 subjects from the analysis according to the inclusion criteria (CGD n = 6, PGD n = 18) for the following reasons: karyotype other than 46,XY (n = 4), diagnosed with another DSD condition (n = 1), possibility of gene defect due to 46XY trans (1;2)(q23;q11.2) (n = 1), or insufficient data on phenotype / serum testosterone / genetics / gonadal histology (n = 18). A patient with a balanced translocation 46,XY,inv(9)(p11;q13), karyotype considered a normal variant, was included in the study.

A small group of patients (n = 10) had typical female genitalia but without a uterus and evidence of gonadal dysfunction from biochemical, genetic, or histological investigations. The absence of a uterus was confirmed by laparoscopy or laparotomy in 6 cases. We included these individuals in the PGDf group, as the phenotype is not consistent with typical CGD, and absence of Mullerian remnants suggested functioning gonads. Therefore, 310 patients were included in the final analysis (CGD n = 100, PGDf n = 107, PGDm n = 103).

### Data Analysis

External masculinization scores (9) and external genital scores (EGS) (10) were calculated from the questionnaire data. The EGS was assessed for patients who presented before 2 years of age. For analysis of biochemical data (LH, FSH, testosterone), each institution's threshold was used. Information on assays for these hormone measurements was not available. In cases where a normal range was not provided (66%-73% each category), normative values from the Canadian Laboratory Initiative on Pediatric Reference Intervals (CALIPER) were used (11). For AMH analysis, each institution used either ELISA or chemiluminescence immunoassay and the age-specific male reference was used for assessment (Beckman Coulter Cat# A73818, RRID:AB_3674123; Beckman Coulter Cat# B13127, RRID:AB_2892998; Roche Cat# 06331076, RRID:AB_2895131).

To evaluate onset of puberty, an age threshold of ≥13.0 years was used, as 90% of males would have developed a testis volume ≥4 mL at that age (12). We evaluated puberty completion in patients who were either older than 17 years of age or had already reached Tanner stage G5 prior to this age, as 95% of males would have reached stage 5 (12).

### Histological Data

Clinicians were asked to classify reported gonadal histology consistent with 1 of the following options: “streak gonad,” “dysgenetic testis” (including undifferentiated gonadal tissue [UGT]), “normal/almost normal testis,” and “replaced by tumor.” Clinicians were also asked to submit anonymized histology reports and pathology slide scans for review if feasible. Members of the study team (M.C., K.V.) with expertise in germ cell pathology categorized histology reports into streak, dysgenetic or regressed gonad, UGT, ovotestis, or normal testis. Pathology slides or slide scans were classified as (1) normal testis; (2) dysgenetic testis: poorly developed seminiferous tubules with increased intertubular stroma with or without the following characteristics: fibrosis, intracapsular growth of testis tubules, diminished tubular diameter, decreased number or absence of germ cells (Sertoli cell only tubules); (3) UGT: germ cells were organized together with Sertoli/granulosa-like cells in cord-like structures or resided without apparent organization in a background of gonadal stromal cells; (4) streak gonad: fibrous stroma devoid of germ cells; cord like structures, when recognizable, were interpreted as UGT that had lost its germ cells and had undergone a fibromatous involution; or (5) gonadal regression: Mullerian and Wolffian duct derivatives only (13).

### Ethics

The I-DSD Registry is approved by the National Research Ethics Service in the United Kingdom as a research database. The State University of Campinas and University of São Paulo have local institutional ethics approvals in place to share anonymized data for research studies.

### Statistics

Data were described as median [first quartile-third quartile] unless specified. Categorical variables were compared using the Fisher exact test and a *P* value of <.05 was considered significant.

## Results

### Patients

**Figure 1.**
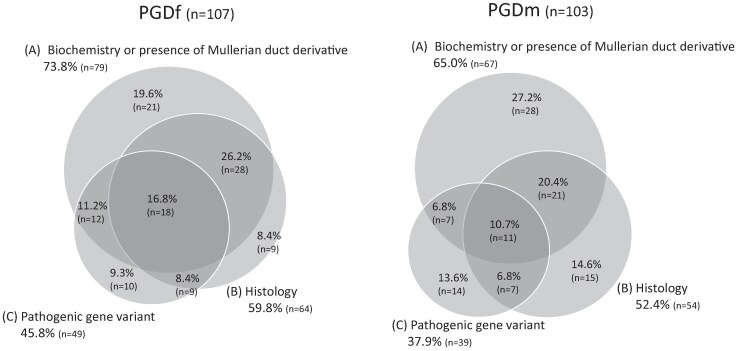
Distribution of inclusion criteria. The Venn diagram shows the number of individuals with PGD who fulfilled the inclusion criteria (A) testosterone levels after hCG stimulation test less than twice the basal value, low basal testosterone with high gonadotropin levels in minipuberty or puberty, AMH or inhibin B levels below male reference range, or presence of Mullerian duct derivatives, (B) histology consistent with GD, and (C) pathogenic variants in genes associated with GD, in addition to (1) 46,XY karyotype and (2) atypical genitalia.

Patients who fulfilled the inclusion criteria comprised 107 individuals with PGDf and 103 with PGDm. In PGDf, in addition to (1) 46,XY karyotype and (2) atypical genitalia, (3A) 74% fulfilled clinical evidence of gonadal dysgenesis, (3B) 60% fulfilled histology criteria, and (3C) 46% fulfilled pathogenic gene variant. In the PGDm category, 65%, 52%, and 38% of the patients fulfilled these criteria, respectively (Fig. 1). The individuals were born between 1941 and 2020 (median 1997 [1987-2006]: Table 2). The patients were from European centers (n = 166, 53.5%), South America (n = 118, 38.1%), and Asia (n = 26, 8.4%). There was no significant difference in gestational age or birth weight between the categories (Table 1). There was a positive family history of DSD in 17.4% of CGD, 19.0% of PGDf and 25.8% of PGDm. A similar proportion also reported a family history of infertility (Table 2).

**Table 2.** Characteristics, presentation of the patients, and internal/external genital features

Parameter	CGD (n = 100)	PGDf (n = 107)	PGDm (n = 103)	*P* value
**Year of birth (range)**	1941-2020	1951-2019	1962-2019	Total data 1941-2020
Median [1st-3rd quartile]	1995 [1986-2000]	1995 [1985-2006]	2002 [1991-2010]	1997 [1987-2006]
**Gestation (weeks)**				
Median [1st-3rd quartile]	39 [38-40] (n = 29)	39 [37.3-40] (n = 30)	38.5 [38-40] (n = 34)	CGD vs PGDf ns CGD vs PGDm ns PGDf vs PGDm ns
Preterm	11.5% (n = 6/52)	11.3% (n = 6/53)	12.8% (n = 10/78)	
Term	88.5% (n = 46/52)	88.7% (n = 47/53)	87.2% (n = 68/78)	
**Birth weight (g)**				
Median [1st-3rd quartile]	3240 [2814-3614] (n = 46)	3290 [2790-3775] (n = 68)	3185 [2800-3500] (n = 81)	CGD vs PGDf ns CGD vs PGDm ns PGDf vs PGDm ns
**Positive family history**				CGD vs PGDf ns PGDf vs PGDm ns CGD vs PGDm ns
DSD	17.4% (n = 15/86)	19.0% (n = 16/84)	25.8% (n = 24/93)	
Infertility	2.3% (n = 2/86)	6.0% (n = 5/84)	5.4% (n = 5/93)	
**Age of the first presentation (y)**	15.0 [8.4-17.9] (n = 94)	1.3 [0.1-13.9] (n = 91)	0.5 [0.1-4.0] (n = 94)	CGD vs PGDf *P* < .00001 CGD vs PGDm *P* < .00001 PGDf vs PGDm *P* = .031
**External genitalia assessed before 24 mo old**	n = 13	n = 53	n = 69	
**Genital tubercle length**	n = 8	n = 34	n = 50	
<10 mm	8 (100%)	9 (26.5%)	3 (6%)	
10-20 mm	0	13 (38.2%)	21 (42%)	
21-25 mm	0	4 (11.8%)	10 (20%)	
26-30 mm	0	5 (14.7%)	10 (20%)	
>31 mm	0	3 (8.8%)	6 (12%)	
**Urethral meatus opening**	n = 12	n = 45	n = 69	
Top of the genital tubercle (3)	1 (8.3%)	2 (4.4%)	8 (11.6%)	
Coronal glandular (2.5)	0	1 (2.2%)	4 (5.8%)	
Along the genital tubercle (2)	0	3 (6.7%)	8 (11.6%)	
At the genital tubercle base (1.5)	0	5 (11.1%)	14 (20.3%)	
Labioscrotal (1)	1 (8.3%)	9 (20.0%)	18 (26.1%)	
Perineal (0)	10 (83.3%)	25 (55.6%)	17 (24.6%)	
**Labioscrotal fusion**	n = 13	n = 48	n = 68	
Fused (3)	0	8 (16.7%)	43 (63.2%)	
Posterior fusion (1.5)	0	16 (33.3%)	13 (19.1%)	
Unfused (0)	13 (100%)	24 (50%)	12 (17.6%)	
**Either gonad**	n = 26	n = 122	n = 140	
Labioscrotal (1.5)	0	16 (13.1%)	52 (37.1%)	
Inguino-labioscrotal (1.0)	0	2 (1.6%)	2 (1.4%)	
Inguinal (0.5)	1 (3.8%)	31 (25.4%)	34 (24.3%)	
Impalpable (0)	25 (96.2%)	73 (59.8%)	52 (37.1%)	
**EGS**				
Median [1st-3rd quartile]	0 [0-0.6] (n = 8)	4.0 [2.0-5.5] (n = 33)	7.0 [5.5-8.3] (n = 51)	CGD vs PGDf <.0001 CGD vs PGDm <.00001 PGDf vs PGDm <.00001
**Presence of uterus**	100% (n = 96/96)	51.0% (n = 52/102)	31.3% (n = 30/96)	PGDf vs PGDm .0062
**Presence of Fallopian tube**	91.5% (n = 65/71)	35.5% (n = 27/76)	18.8% (n = 16/85)	CGD vs PGDf <.00001 CGD vs PGDm <.00001 PGDf vs PGDm .0003
	1.4% (n = 1/71) blind ending in the right			

Data presented as median [1st-3rd quartile], NS-not significant. The number of patients available for each analysis are less than the group totals because of missing data.

Abbreviations: CGD, complete gonadal dysgenesis; DSD, differences of sex development; EGS, external genital score; PGDf, partial gonadal dysgenesis assigned female; PGDm, partial gonadal dysgenesis assigned male.

### Presentation

**Figure 2.**
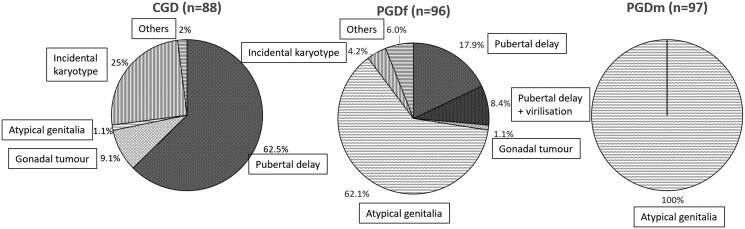
Mode of clinical presentation (%). In CGD, 62.5% presented with pubertal delay, followed by incidental karyotype findings and gonadal tumor symptoms such as abdominal pain/discomfort and mild fever. In PGD, the majority presented with atypical genitalia. In PGDf, 17.5% presented with pubertal delay, 8.2% presented with pubertal delay and virilization such as clitoral enlargement and hirsutism.

The individuals with CGD presented at age 15.0 [8.4-17.9] years, with the majority (62.5%) complaining of delayed puberty (Fig. 2, Table 2). A diagnosis at an earlier age was obtained in 25.0%, where associated features led to karyotype analysis. Symptoms caused by the effects of gonadal tumor such as abdominal pain led to the diagnosis of CGD in 9.1% (n = 8/88) at age 12.7 (range 7.8-16.6) years. In contrast, individuals with PGD presented at a younger age (PGDf 1.3 [0.1-13.9] years; PGDm 0.5 [0.1-4.0] years) due to atypical genitalia in all patients with PGDm and 62.1% in PGDf. Delayed puberty was the presenting feature in 17.9% of PGDf patients and in 8.4%, it was associated with virilization.

### Phenotype

In individuals who presented before age 2 years, the external genital appearance was more masculinized in PGDm compared to PGDf (EGS 7.0 [5.5-8.3] vs 4.0 [2.0-5.5]; *P* < .0001) (Table 2). A uterus was present in a greater proportion of PGDf individuals compared with PGDm (51.0% vs 31.3%, *P* = .006) and in all patients with CGD as per case definition. Fallopian tubes were present in 91.5% of individuals with CGD, whereas in PGDf and PGDm, fallopian tubes were present in 35.5% and 18.8%, respectively.

### Comorbidities

A high frequency of comorbidities was reported in all categories with no significant differences between groups (CGD 38.0%, PGDf 33.0%, PGDm 33.3%). Detailed comorbidities are listed in Supplementary Table S1 (14). Examples of neurodiversity such as developmental delay, learning difficulties, attention deficit hyperactivity disorder, autistic spectrum disorder, and structural brain anomalies were the most frequent (CGD 28.9%, PGDf 21.3%, PGDm 26.3%). The next most frequent group was renal disorders including Wilms’ tumor, congenital proteinuria, and structural anomalies (CGD 23.7%, PGDf 12.8%, PGDm 31.6%). While not all patients had the *WT1* gene screened, 83% (n = 10/12) of individuals with *WT1* mutation had associated renal comorbidities.

### Biochemical Markers of Gonadal Function

#### Minipuberty (age 0-6 months)

There was no significant difference in basal serum testosterone levels in individuals with PGDm and PGDf (Table 3). FSH levels were similar between PGDf and PGDm and above the reference range in all individuals with CGD and 67.9% in PGDf and 64.7% in PGDm. LH levels were similar in the groups and above the reference range in 16.7% CGD, 7.1% PGDf, and 17.6% PGDm (Table 3).

**Table 3.** Biochemical data at the first presentation

	CGD (n = 100)	PGDf (n = 107)	PGDm (n = 103)	*P* value
**Basal testosterone 0-6 mo (nmol/L)**				
Median [1st-3rd quartile]	0.30 [0.095-0.82] (n = 6)	1.56 [0.45-3.52] (n = 23)	2.43 [0.69-4.88] (n = 31)	CGD vs PGDf 0.002 CGD vs PGDm <.00001 PGDf vs PGDm ns
Range	0.087-1.1	0.006-9.6	0.07-8.5	
**LH 0-6 mo (IU/L)**	n = 6	n = 28	n = 34	
Median [1st-3rd quartile]	3.25 [1.65-5.6]	2.8 [1.6-4.9]	4.25 [1.25-8.8]	CGD vs PGDf ns CGD vs PGDm ns PGDf vs PGDm ns
Range	0.1-30	0.1-18.2	0.1-97.1	
**LH elevated: 0-6 mo** *^a^*	16.7% (n = 1/6)	7.1% (n = 2/28)	17.6% (n = 6/34)	CGD vs PGDf ns CGD vs PGDm ns PGDf vs PGDm ns
**FSH 0-6 months (IU/L)**	n = 6	n = 28	n = 34	
Median [1st-3rd quartile])	28.7 [20.6-38.4]	6.7 [3.3-15.6]	6.8 [2.75-12.1]	CGD vs PGDf ns CGD vs PGDm ns PGDf vs PGDm ns
Range	11.5-120	0.1-57	0.7-28.5	
**FSH elevated: 0-6 mo** *^a^*	100% (n = 6/6)	67.9% (n = 19/28)	64.7% (n = 22/34)	CGD vs PGDf ns CGD vs PGDm ns PGDf vs PGDm ns
**Basal testosterone ≥13 y (nmol/L)**	n = 44	n = 17	n = 14	
Median [1st-3rd quartile]	0.8 [0.49-1.18]	3.3 [1.08-11.24]	0.83 [0.49-4.04]	CGD vs PGDf 0.009 CGD vs PGDm .00003 PGDf vs PGDm ns
Range	0-4.56	0.45-22.62	0.49-20.72	
**LH ≥13 y (IU/L)**	n = 65	n = 35	n = 13	
Median [1st-3rd quartile]	27.0 [17.0-37.8]	18.0 [9.9-31.8]	15.8 [10.0-21.0]	CGD vs PGDf .027 CGD vs PGDm ns PGDf vs PGDm ns
Range	0.16-112.8*^b^*	0.01-67.8	0.9-149	
**LH elevated (≥13 y)** *^a^*	93.8% (n = 61/65)	77.1% (n = 27/35)	76.9% (n = 10/13)	CGD vs PGDf .022 CGD vs PGDm .086 PGDf vs PGDm ns
**FSH ≥13 y (IU/L)**	n = 65	n = 35	n = 13	
Median [1st-3rd quartile]	85.0 [58.0-119.9]	52.8 [29.5-78.1]	48.9 [44.0-70.0]	CGD vs PGDf .00006 CGD vs PGDm .0003 PGDf vs PGDm ns
Range	0.7-184.8*^b^*	2.4-170.0	18.1-100.0	
**FSH elevated** *^a^* **(≥13 y)**	95.4% (n = 62/65)	80.0% (n = 28/35)	100% (n = 13/13)	CGD vs PGDf .030 CGD vs PGDm ns PGDf vs PGDm ns
**hCG stimulated testosterone** *^c^* **(nmol/L)**	n = 15	n = 34	n = 46	
Median [1st-3rd quartile]	0.56 [0.4-1.04]	1.98 [0.59-9.70]	3.6 [1.04-7.29]	CGD vs PGDf ns CGD vs PGDm .0026 PGDf vs PGDm .075
Range	0.087-11	0.035-28.8	0.007-19	
**hCG stimulated testosterone** *^c^*				
Doubled	20% (n = 3/15)	47.2% (n = 17/36)	66.0% (n = 31/47)	CGD vs PGDf ns CGD vs PGDm .003 PGDf vs PGDm .075
**AMH** *^d^*				
Low	95.7% (n = 22/23)	48.0% (n = 12/25)	58.1% (n = 18/31)	CGD vs PGDf .0003 CGD vs PGDm .002 PGDf vs PGDm ns
Normal	4.3% (n = 1/23)	52.0% (n = 13/25)	41.9% (n = 13/31)	
**Inhibin B**				
Low	100% (n = 20/20)	66.7% (n = 8/12)	69.2% (n = 9/13)	CGD vs PGDf .014 CGD vs PGDm .016 PGDf vs PGDm ns
Normal	0% (n = 0/20)	33.3% (n = 4/12)	30.8% (n = 4/13)	

Data presented as median [1st-3rd quartile]. The number of patients available for each analysis are less than the group totals due to missing data.

Abbreviations: CGD, complete gonadal dysgenesis; NS, not significant; PGDf, partial gonadal dysgenesis assigned female; PGDm, partial gonadal dysgenesis assigned male.

^*a*^Institution's threshold or CALIPER cutoff was used. LH: institution's reference provided in n = 24/87, 31/93, 24/85 in CGD, PGDf and PGDm, respectively (28%-33%). FSH institution's reference provided in n = 24/87, 32/93, 23/85 in CGD, PGDf, and PGDm, respectively (27%-34%). CALIPER cutoff: LH >10 IU/L, FSH >4 IU/L at 0-6 months; LH >8 IU/L, FSH > 7 IU/L at >13 y.

^*b*^Individuals with CGD with low LH and FSH were associated with gonadal tumor.

^*c*^Taken at any age.

^*d*^Based on age appropriate male anti-Mullerian hormone reference range in each institution.

#### Age ≥13 years

In patients who presented at age ≥13 years for the first time, basal serum testosterone levels were similar in PGDf and PGDm (Table 3). LH and FSH levels were also similar in these groups (Table 3). A greater proportion of individuals with CGD had elevated serum LH levels compared to other groups (93.8% in CGD vs 77.1% in PGDf, *P* = .022; 76.9% in PGDm, *P* = .086). FSH was above the reference range in 95.4% CGD, 80% PGDf, and 100% PGDm in this age group (CGD vs PGDf *P* = .030, CGD vs PGDm *P* = ns, PGDf vs PGDm *P* = ns). Individuals with CGD with low LH and FSH levels were associated with gonadal tumor.

There was no significant difference in day 4 serum testosterone levels after hCG stimulation (taken at any age) between PGDf and PGDm (Table 3). In these groups, a similar proportion of individuals had low levels of AMH and inhibin B (48% and 58.1% low in AMH and 66.7% and 69.2% low in inhibin B, respectively: Table 3).

### Genetics

Genetic screening was performed in 78.7% of the cohort with various methods; targeted gene screening (71%), gene panels (13%), and whole exome sequencing (15%). This identified the likely genetic cause (gene or chromosome alterations) in 42.3% of the cohort (n = 131/310). Of those who underwent screening, 48.3% of individuals with CGD, 59.0% with PGDf, and 50.0% with PGDm had a gene or chromosome alteration identified. The most frequent gene alterations in CGD were *SRY* (23.6%; n = 21/89) and *WT1* (9.0%; n = 8/89). In PGD, alterations in *NR5A1* were the most frequent finding (PGDf: 42.2%; n = 35/83; PGDm: 25.6%; n = 20/78). Gene alterations are listed in Supplementary Table S2 (15).

### Pubertal Development

#### PGD male gender

**Figure 3.**
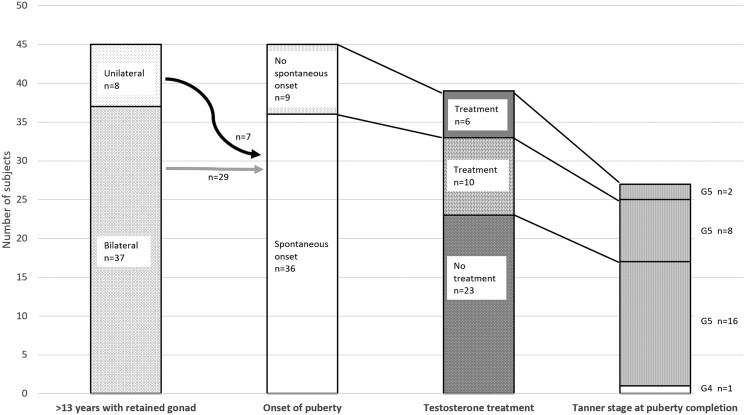
Pubertal outcome in individuals with PGD assigned male. There were 45 individuals with PGD assigned male aged ≥13 years with unilateral (n = 8) or bilateral (n = 37) gonads in the cohort. Spontaneous onset of puberty was observed in 80% (n = 36/45; n = 7 unilateral, n = 29 bilateral gonad). In individuals with spontaneous puberty onset, 30.3% (n = 10/33) required testosterone treatment to complete puberty. Data on whether testosterone treatment was given were missing in 6 individuals. At the assessment of puberty completion, 94% (n = 16/17) of the individuals who did not receive testosterone treatment attained Tanner G5 spontaneously. All patients who received testosterone treatment attained Tanner G5. Data on the final Tanner stage were available in 27 individuals.

Information on puberty was available in 45 individuals with PGD who assigned male at age ≥13 years, including 9 individuals reassigned from female to male before age 13 years, with at least 1 retained gonad (bilateral gonads n = 37, unilateral n = 8). More than half of the individuals in this group (55.6%, n = 25/45) had at least 1 gonad at the labioscrotal region at the first presentation and 44% (n = 20/45) underwent unilateral or bilateral orchidopexy. Spontaneous onset of puberty (Tanner stage G2 or testis volume ≥4 mL) occurred in 80.0% (n = 36/45) at age 12 [10.6-12.0] years. Of these, 30.3% (n = 10/33) were treated with testosterone to complete puberty (Fig. 3). The decision on whether to start testosterone treatment was taken by individual clinicians. In puberty completion assessment, 59.3% (n = 16/27) achieved Tanner G5 without hormone treatment at age 18.1 [16.0-20.0] years. In individuals who had spontaneous puberty completion, the Tanner staging was: G5 n = 16 (94.1%) and G4 n = 1 (5.9%), the median testis volume was 8.0 [4.8-8.5] mL (n = 12 gonads) and the stretched penile length was 6.8 [5.9-8.0] cm (n = 10) (Table 4). Serum LH and FSH levels were elevated in 94.4% (n = 17/18) and 100% (n = 18/18), respectively.

**Table 4.** PGD male pubertal development, surgical outcome and sex reassignment

**Information on PGD assigned male at age 13 y with at least 1 retained gonad**	n = 45 (bilateral gonads n = 37, unilateral n = 8)
**Spontaneous onset of puberty**	80.0% (n = 36/45)
**Age of spontaneous onset of puberty**	12.0 [10.6-12.0] (n = 26)
**Patients with spontaneous onset of puberty who received testosterone treatment**	30.3% (n = 10/33)
**Frequency of achieving Tanner G5 spontaneously** *^a^*	59.3% (n = 16/27)
**Puberty assessment of the patients with spontaneous puberty who received no testosterone treatment at completion**	Age 18.1 [16.0-20.0] y (n = 13)
	Tanner stage: G5 94.1% (n = 16/17), G4 5.9% (n = 1/17)
	Pubic hair stage: PH5 100% (n = 8/8)
	Testicular volume (mL): 8.0 [4.8-8.5] (n = 12 gonads)
	Stretched penile length (cm): 6.8 [5.9-8.0] (n = 10)
	Gynecomastia: n = 2/16 (12.5%)
**Biochemistry data of the patients with spontaneous puberty who received no testosterone treatment at puberty completion**	Median age 17.8 [15.3-19.4] years (n = 16)
	Basal testosterone 20.0 [14.3-29.2] nmol/L (n = 16)
	LH 13.2 [9.5-21.5] IU/L (n = 16)
	FSH 28.4 [16.6-49.5] IU/L (n = 16)
**Orchidopexy (>13 y)** *^b^*	
Unilateral	30.2% (n = 16/53)
Bilateral	18.9% (n = 10/53)
**Hypospadias repair procedure (>13 years)** *^b^*	
Once	30.2% (n = 16/53)
Twice	15.1% (n = 8/53)
More than twice	28.3% (n = 15/53)
**Sex reassignment (male to female)** *^c^*	5.3% (n = 5/94)
**Age of sex reassignment**	Infancy (0-1 y) n = 2
	Childhood (1-9 y) n = 1
	Adolescent (10-19 y) n = 2
**Frequency of sex reassignment in geographical regions**	Europe 7.9% (n = 3/38)
	South America 3.7% (n = 2/54)
	Asia 0% (n = 0/11)

Data presented as median [1st-3rd quartile]. The number of patients available for each analysis are less than the group totals due to missing data.

Abbreviations: NS, not significant; PGDf, partial gonadal dysgenesis.

^*a*^The numbers include subjects who had an assessment at age >17 years or had attained G5 prior to this. Subjects without information on final Tanner stage were excluded from the denominator.

^*b*^The denominator includes patients who had bilateral gonadectomy before age 13 y.

^*c*^Incudes prepubertal individuals.

Factors associated with spontaneous puberty were explored. The external masculinization scores, EGS, or serum basal testosterone levels at the first investigation were not predictive (Supplementary Table S3 (16)). However, patients who developed spontaneous puberty were more likely to have at least 1 labioscrotal gonad at presentation compared to those with induced puberty (62.9%; n = 22/35 vs 12.5%; n = 1/8, *P* = .017). Also, a higher proportion of individuals in the spontaneous puberty group had a testosterone response to hCG more than double the basal level (88%; n = 15/17, vs 33%; n = 1/3, *P* = .087). All individuals with gene alterations in *NR5A1* with gonads (n = 14; 78.6% uni-/bilateral labioscrotal gonad at the first presentation) had spontaneous onset of puberty compared to only 50% (n = 4/8; 62.5% uni-/bilateral labioscrotal gonad at the first presentation) of the individuals with other gene mutations (*P* = .078). Furthermore, 15.4% (n = 2/13) of the patients with *NR5A1* gene alteration required subsequent testosterone treatment (1 individual started at age 15 years and the other age unknown).

Data beyond puberty (n = 16, age ≥19 years [median age 21.5; range 19-50 years]) showed that patients who completed puberty without testosterone treatment did not require further replacement therapy.

#### CGD

In women with CGD who presented aged ≥13 years, spontaneous breast development was noted in 18.5% (n = 12/65) (Table 5). Puberty induction was started at age 16 [14.0-17.3] years (n = 66) and 72.3% (n = 34/47) of the individuals aged ≥15 years achieved menarche (Table 5). The median age of menarche was 17.3 [16.5-19.0] years (n = 19), following induction treatment of 2.3 [0.7-4.0] years (n = 17).

**Table 5.** Puberty development, surgical outcome and sex reassignment in CGD and PGD assigned female

Individuals with gonad in situ (>13 y)	CGD (n = 68)	PGDf (n = 32)
**Breast development**	18.5% (n = 12/65)	30.8% (n = 8/26)
	B2 n = 4, B3 n = 1, B4 n = 4, B5 n = 1, unknown n = 2	B2 n = 3, B3 n = 3, B5 n = 2
		n = 6/8 also had virilization
**Virilization**	0% (n = 0/65)	42.3% (n = 11/26)
		hirsutism n = 5, clitoral enlargement n = 7, “virilization” n = 1
**Association with gonadal tumor**	66.7% (n = 8/12) of breast development associated with tumor (gonadoblastoma, seminoma, dysgerminoma, mixed germ cell tumor)	25% (n = 2/8) of breast development associated with tumor; 36.4% (n = 4/11) of virilization associated with tumor (gonadoblastoma, dysgerminoma, seminoma)
**Age of puberty induction (y)**	16.0 [14.0-17.3] (n = 66)	13.0 [11.0-17.0] (n = 51)
**Menarche achieved (at >15 y)**	72.3% (n = 34/47)	30.0% (n = 9/30)
**Clitoral reduction surgery** *^a^*	0% (n = 0/82)	47.1% (n = 33/70)
**Vaginoplasty** *^a^*	0% (n = 0/81)	44.9% (n = 31/69)
**Sex reassignment (female to male)** *^a^*	0% (n = 0/86)	16.1% (n = 15/93)
**Age of sex reassignment**	N/A	Infancy (0-1 y) n = 3
		Childhood (1-9 y) n = 6
		Adolescent (10-19 y) n = 4
		Adult (20 y) n = 1
		Unknown n = 1
**Frequency of sex reassignment in geographical regions**	N/A	Europe 18.2% (n = 6/33)
		South America 12.7% (n = 8/63)
		Asia 9.1% (n = 1/11)

Data presented as median [1st-3rd quartile]. The number of patients available for each analysis are less than the group totals due to missing data.

Abbreviations: CGD, complete gonadal dysgenesis; N/A, not available; PGDf, partial gonadal dysgenesis assigned female.

^*a*^Includes prepubertal individuals.

#### PGD female gender

Of individuals with PGD assigned female at age ≥13 years with at least 1 gonad (n = 18/22 individuals with intra-abdominal or impalpable gonad, n = 4/22 uni-/bilateral inguinal gonad) 42.3% (n = 11/26) developed features of virilization (hirsutism n = 5, clitoral enlargement n = 7) (Table 5) and had testosterone levels appropriate for boys (9.3 [7.2-13.1] nmol/L, n = 6). Spontaneous breast development was noted in 30.8% (n = 8/26; Tanner stage B2 n = 3, B3 n = 3, B5 n = 2). Among this group, 6 patients (75%) presented with virilization along with breast development. Two individuals with virilization were treated with a GnRH analogue. Puberty induction with oestrogen was started at age 13.0 [11.0-17.0] years (n = 51) and 30% (n = 9/30) achieved menarche assessed at age >15 years.

### Surgical Interventions

#### Genital surgery

In PGDf, 47.1% (n = 33/70) of individuals underwent clitoral reduction surgery and 44.9% (n = 31/69) had vaginoplasty (Table 5). About half (n = 26/53) and 73.6% (n = 39/53) of the individuals with PGDm underwent orchidopexy and hypospadias repair, respectively (Table 4).

#### Gonadal biopsy

Gonadal biopsy was performed in 26.0% (n = 19/73) of individuals with CGD; all subsequently had gonadectomy (Table 6). In PGDf, biopsy was performed in 22.9% (n = 19/83), and 52.6% (n = 10/19) subsequently had gonadectomy. Of those who had retained gonads, 5 individuals reassigned to male sex. In PGDm, 43.5% of the individuals had unilateral or bilateral gonad biopsy (n = 40/92; n = 12 unilateral, n = 28 bilateral) at median age 1.8 years [1.0-4.5] which led to subsequent gonadectomy in 22.5% (n = 9/40).

**Table 6.** Biopsy, gonadectomy, and histology

	CGD (n = 100)	PGDf (n = 107)	PGDm (n = 103)	
**Biopsy**				
Unilateral	4.1% (n = 3/73)	0%	13.0% (n = 12/92)	
Bilateral	21.9% (n = 16/73)	21.7% (n = 18/83)	30.4% (n = 28/92)	
Unknown	0%	1.2% (n = 1/83)	0%	
Median age [1st-3rd quartile]	11.0 [4.2-15.4] (n = 17)	1.8 [0.5-3.7] (n = 16)	1.8 [1.0-4.5] (n = 39)	
**Gonadectomy**				
Bilateral	93.6% (n = 88/94)	75.0% (n = 75/100)	23.5% (n = 23/98)	
Unilateral	0%	0%	19.4% (n = 19/98)	
No	6.4% (n = 6/94)	25.0% (n = 25/100)	57.1% (n = 56/98)	
**Age of gonadectomy (y)**			Bilateral gonadectomy	Unilateral gonadectomy
Median [1st-3rd quartile]	16.0 [13.1-18.3] (n = 82)	6.5 [1.6-15.1] (n = 68)	4.0 [3.0-14.6] (n = 23)	2.7 [0.87-4.8] (n = 19)
Range	0.42-45	0.08-43	1.5-34	0.08-14
Infancy (0-1 yr)	1.3% (n = 1/79)	18.2% (n = 12/66)	4.8% (n = 1/21)	28.6% (n = 4/14)
Childhood (1-9 y)	22.8% (n = 18/79)	51.5% (n = 34/66)	57.1% (n = 12/21)	64.3% (n = 9/14)
Adolescent (10-19 y)	63.3% (n = 50/79)	25.8% (n = 17/66)	33.3% (n = 7/21)	7.1% (n = 1/14)
Adult (>20 y)	16.5% (n = 13/79)	7.6% (n = 5/66)	4.8% (n = 1/21)	0% (n = 0/14)
**Gonadectomy findings: clinicians’ multiple-choice question (per gonad)**				
Streak gonad	75.7% (n = 87/115)	19.0% (n = 24/126)	28.6% (n = 16/56)	
Dysgenetic testis	7.0% (n = 8/115)	51.6% (n = 65/126)	35.7% (n = 20/56)	
Normal/almost normal testis	0%	18.3% (n = 23/126)	1.8% (n = 1/56)	
Replaced by tumor	9.6% (n = 11/115)	2.4% (n = 3/126)	1.8% (n = 1/56)	
Ovary	2.6% (n = 3/115)	0%	0	
Ovotestis	0%	0%	1.8% (n = 1/56)	
Gonadal regression	5.2% (n = 6/115)	8.7% (n = 11/126)	30.4% (n = 17/56)	
**Histology report overview**	n = 9 gonads from 5 patients	n = 17 gonads from 11 patients	n = 8 gonads from 5 patients	
Streak ± UGT	55.6% (n = 5/9)	29.4% (n = 5/17)	12.5% (n = 1/8)	
Dysgenetic	11.1% (n = 1/9)	35.3% (n = 6/17)	62.5% (n = 5/8)	
Testis	0%	11.8% (n = 2/17)	0%	
Absent gonad	0%	0%	12.5% (n = 1/8)	
Not described	33.3% (n = 3/9)	17.6% (n = 3/17)	12.5% (n = 1/8)	
**Whole slide overall features (per gonad)**	n = 10 gonads from 5 patients	n = 7 gonads from 5 patients	n = 11 gonads from 7 patients	
Streak gonad	30% (n = 3/10 [n = 1 +UGT])	14.3% (n = 1/7)	9.1% (n = 1/11)	
Dysgenetic testis	0%	85.7% (n = 6/7 [n = 2 +UGT])	63.6% (n = 7/11)	
Gonadal regression	60% (n = 6/10)	0%	0%	
UGT	10% (n = 1)	0%	0%	
Normal testis	0%	0%	27.3% (n = 3/11 [n = 1 +UGT])	
**Whole slide testicular dysgenesis features**	N/A	Reduced tubular diameter n = 1, Irregular tubular shape n = 2, Intracapsular growth n = 3, SCO tubules n = 2, Reduced GC number n = 4, Increased interstitium n = 2	Thickened BM n = 4, SCO tubules n = 4, LC hyperplasia n = 2, Reduced GC number n = 2, Intracapsular growth n = 2, Interstitial fibrosis n = 3, Increased intertubular space n = 1	
**Pre-/malignancy (per individual)**				
Unilateral	18.1% (n = 15/83)	4.5% (n = 3/66)	5.9% (n = 2/34)	
Bilateral	15.7% (n = 13/83)	15.2% (n = 10/66)	2.9% (n = 1/34)	
**Type of malignancy/premalignancy (per gonad)**	n = 41	n = 23	n = 3	
	Gonadoblastoma 19 (46.3%)	Gonadoblastoma 12 (52.2%)	Mixed GCT 1 (33%)	
	Germinoma + GB 1 (2.4%)	Dysgerminoma/seminoma 5 (21.7%)	Gonadoblastoma 1 (33%)	
	Dysgerminoma (±GB) 13 (31.7%)	Sex cord stromal tumor 3 (13.0%)	GCNIS 1 (33%)	
	Mixed GCT 2 (4.9%)	GCNIS 5 (21.7%)		
	GCNIS 2 (4.9%)			
	Yolk sac tumor 1 (2.4%)			
	Seminoma 1 (2.4%)			
	Unknown 2 (4.9%)			
**Age of malignancy/premalignancy diagnosis** *^a^*	Seminoma 16.8, 15.3 y (n = 2)	Seminoma 18.3 y (n = 1)	Mixed GCT 19 y (n = 1)	
Median age [range], y	Dysgerminoma 15.0 [9.6-21.2] (n = 9)	Dysgerminoma 20 y, unknown (n = 2)	Gonadoblastoma 2.5 y (n = 1)	
	Germinoma + GB 18.9 yrs (n = 1)	Sex cord stromal tumor 6.4, 9.8 y (n = 2)	GCNIS 8.16 y (n = 1)	
	Mixed GCT 7.8, 16.5 y (n = 2)	Gonadoblastoma 2.5[0.1-17] (n = 6)		
	Yolk sac tumor 13.5 y (n = 1)	GCNIS 3.8 y (n = 2)		
	Gonadoblastoma 16.5 [0.4-24.8] (n = 11)			
	GCNIS 3.8 y (n = 1)			

Data presented as median [1st-3rd quartile]. The number of patients available for each analysis are less than the group totals due to missing data.

Abbreviations: BM, basement membrane; CGD, complete gonadal dysgenesis; GB, gonadoblastoma; GC, germ cell; GCNIS, germ cell neoplasia in situ; GCT, germ cell tumor; LC, Leydig cell; N/A, not available; PGDf, partial gonadal dysgenesis assigned female; PGDm, partial gonadal dysgenesis assigned male; SCO, Sertoli cell only; UGT, undifferentiated gonadal tissue.

^*a*^Individuals with 2 different tumors were counted for more severe form of tumor.

#### Gonadectomy

Bilateral gonadectomy was performed in 93.6% of individuals with CGD (n = 88/94), and 75% (n = 75/100) of the individuals with PGDf, at ages 16.0 [13.1-18.3] years and 6.5 [1.6-15.1] years, respectively (Table 6). The interval between first presentation and gonadectomy was 0.63 [0.3-1] years (n = 73) in CGD and 1.42 [0.4-4.4] years (n = 51) in PGDf. In PGDm, 19.4% of individuals (n = 19/98) had unilateral and 23.5% (n = 23/98) had bilateral gonadectomy at age 2.7 [0.87-4.8] (n = 19) and 4.0 [3.0-14.6] (n = 23) years, respectively (Table 6). In PGDm, all excised gonads were located in the abdomen; about 60% were regressed or streak gonads.

### Histology of Gonads

Histology diagnosis was reported for 63 patients with CGD, 70 with PGDf, and 40 with PGDm. In addition, our expert panel reviewed detailed histology reports on 21 individuals (34 gonads) and whole slides from 17 (28 gonads).

#### Clinicians’ report

In CGD, “streak gonad” was the most common reported histological feature (75.7%) followed by “replaced by tumor” (9.6%) and “dysgenetic testis” (7.0%). In PGD, “dysgenetic testis” was more common (PGDf: 51.6%, PGDm: 35.7%) than “streak gonad” (PGDf 19.0%, PGDm 28.6%) (Table 6).

#### Histology report review

A similar pattern was found from review of the histology reports. Streak gonad, with or without UGT (55.6%; n = 5/9), was more frequent compared to dysgenetic testis (11.1%; n = 1/9) in CGD and dysgenetic testis was more common in PGD (35.3%; n = 6/17 in PGDf, 62.5%; n = 5/8 in PGDm) (Table 6).

#### Histology slide review

**Figure 4.**
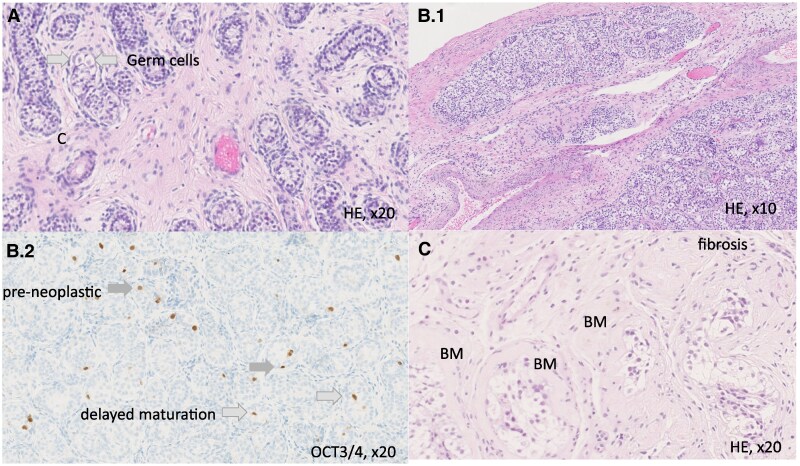
Histology of dysgenetic testis. (A) PGDf (hemizygous *NR5A1* deletion), gonadectomy performed aged 6 years. This shows increased interstitial stroma, reduced tubular diameter, mostly Sertoli cell only tubules. Arrows indicate germ cells. (B) PGDf (*MAP3K1* defect) aged 22 months. (B.1) Intracapsular growth. OCT3/4 staining (B.2) shows peripheral preneoplastic cells (gray arrow) and luminal OCT3/4-positive delayed maturation in white arrow. (C) PGDm (unknown genetic cause). Some tubules and the basement membrane (BM) are extremely thickened and interstitial fibrosis is observed.

The common histology features were regressed gonads (60%, n = 6/10), streak gonads (30%, n = 3/10), and UGT (10%, n = 1/10) in CGD. In PGDf, 85.7% were dysgenetic testis (n = 6/7) with features of UGT in 33.3% (n = 2/6) and 14.3% (n = 1/7) were streak gonad. In PGDm, dysgenetic testis (63.6%, n = 7/11), normal testis (27.3%, n = 3/11; of these n = 1 showed UGT), and streak gonad (9.1%, n = 1/11) were observed. Features of dysgenetic testis noted were reduced tubular diameter, thickened basal membrane, increased interstitial stroma, intracapsular growth, irregular shaped tubules, Leydig cell micronodules or hyperplasia, Sertoli cell only tubules, reduced germ cell number, absent spermatogenesis, and tubular atrophy (Fig. 4, Table 6).

#### Gonadal tumors

Gonadal malignancy or premalignancy (germ cell neoplasia in situ) was reported in 33.7% (n = 28/83; 18.1% unilateral, 15.7% bilateral) in CGD, 19.7% (n = 13/66; 4.5% unilateral, 15.2% bilateral) in PGDf, and 8.8% (n = 3/34; 5.9% unilateral, 2.9% bilateral) in PGDm (CGD vs PGDf, *P* = .066; CGD vs PGDm, *P* = .005). The common types of tumors in all 3 groups were gonadoblastoma (47.8%, n = 32/67), dysgerminoma/seminoma (28.4%, n = 19/67), and germ cell neoplasia in situ (11.9%, n = 8/67). Other examples included mixed germ cell tumor (4.5%, n = 3/67), sex cord stromal tumor (4.5%, n = 3/67), and yolk sac tumor (1.5%, n = 1/67) (Table 6).

### Sex Reassignment

Five individuals (n = 5/94; 5.3%) with PGDm were reassigned to female during infancy (n = 2), childhood (n = 1), or adolescence (n = 2) (Table 4). Fifteen individuals (n = 15/93; 16.1%) with PGDf were reassigned to male (infancy n = 3, childhood n = 6, adolescent n = 4, adult n = 1, unknown n = 1) (Table 5).

### Gonadal Tumors and Breast Development

In patients with CGD who had spontaneous breast development, gonadal tumors were found in 67% (n = 8/12; seminoma/dysgerminoma n = 4, mixed germ cell tumor n = 2, gonadoblastoma n = 2). The risk for gonadal tumors was considerably higher in those with spontaneous breast development compared with those without (67% vs 26% [n = 11/42], *P* = .014, odds ratio = 5.6).

In PGDf, 2 individuals with breast development had gonadal tumors (25%; n = 2/8; bilateral gonadoblastoma, bilateral dysgerminoma), and the risk was not greater than other PGDf patients (30%; n = 4/13).

## Discussion

The current study provides detailed characteristics in a large cohort of 46,XY PGD individuals and highlights the wide spectrum of this condition. Key observations show that spontaneous onset of puberty occurred in 80% of individuals raised male and 59% achieved Tanner G5 without hormone treatment. In PGD assigned female at puberty, 42% individuals developed features of virilization.

As 46,XY PGD is a heterogeneous disorder without clearly defined diagnostic criteria, we used the diagnostic criteria from a previous study which requires evidence of testicular dysgenesis, either in a biochemical test (e.g., low testosterone response after hCG stimulation, low AMH or inhibin B), histology findings, or a genetic defect associated with GD (8). As we used CGD as a robust comparator, individuals who did not fit in the clinical picture of typical CGD, such as mild virilization at any age, were classified as PGDf. A small group of individuals with female external genitalia who lacked Mullerian duct structures were difficult to classify. In these cases, absence of uterus was confirmed by laparoscopy in the majority. The variable phenotype likely represents the spectrum of disorder. However, as typical CGD has Mullerian remnants (17), and absence of a uterus suggests AMH secretion, we included this group as PGDf. The study design to include CGD patients was instrumental in illustrating the broad range of phenotypes for 46,XY GD.

A genetic cause was identified in approximately half the study cohort, which is higher than in recent reports (22%-40%) (4, 18). Our results were based on historical data with less robust variant classification and possibly a selection bias which may explain the higher rates. Furthermore, standard karyotyping techniques may miss mosaicism such as 46,XY/45,X. It is possible that some of our cohort may have 45,X cell lines as some comorbidities observed, such as short stature, horseshoe kidney, and coarctation of aorta are also features of 46,XY/45,X mosaicism. This suggests the need for future studies in larger cohorts with more extensive genetic analyses. Nevertheless, the common gene alterations we observed were in accordance with previous smaller studies (18).

Disturbed development of gonads increases the risk of germ cell neoplasia (19). The incidence of gonadal neoplasia in CGD observed in this study (33.7%) is similar to other reports (15%-40% (13, 20)). The prevalence of germ cell neoplasia in PGD is reported to be variable (16%-30% (21)). We noted a lower incidence in PGDf (19.7%) as compared to CGD, which may be due to multiple factors such as earlier gonadectomy (age 6.4 years vs 16 years in CGD), higher levels of “testicularization” of gonads, and an extra-abdominal location (22). In PGDm, there were only 3 cases of neoplasia reported. However, there was significant variation in monitoring practice amongst centers and only one third (n = 34/92) of individuals with PGDm had histology assessment. A collaborative long-term follow-up study, ideally including postpuberty biopsy with histology review undertaken centrally in an expert center, is required to achieve a more accurate risk estimation in boys with PGD.

The frequency of spontaneous or natural completion of puberty was considerably high in our cohort of PGD assigned male, with at least 1 retained gonad (80% had spontaneous onset and 59% completed puberty spontaneously). Two previous studies from Brazil with limited sample size reported discrepant rates of spontaneous puberty in 46,XY PGD (6, 21). Andrade et al reported higher rates; all (n = 10/10) had spontaneous onset and only 1 individual required testosterone treatment, with normal testosterone levels during puberty but with elevated gonadotropin levels despite relatively low hCG-stimulated testosterone levels (<0.3 to 1.7 nmol/L) compared to the present study (median 3.6 nmol/L) (21). Gomes et al reported 57% (n = 12/21) went through puberty spontaneously (6) However, this study included patients assigned female and assessed for puberty from a relatively young age of 9.1 years (6). We found that labioscrotal position of gonad and higher hCG-stimulated testosterone levels are associated with spontaneous pubertal development. Such markers of testis function could be useful when discussing sex assignment of infants. However, EGS at presentation was not related and it is possible that variation in the phenotype as well as limited sample size might explain the lack of correlation. The high rates of spontaneous puberty in patients with *NR5A1* gene alterations confirms findings found in smaller studies (23, 24). A plausible explanation is that steroidogenesis in adult Leydig cells is less dependent on *SF-1/NR5A1* compared to fetal Leydig cells (25). We noted high rates of virilization (44%) with hirsutism and clitoral enlargement in individuals with PGDf who presented with primary amenorrhoea, suggesting residual gonadal function. Interestingly, one third displayed breast development (Tanner 2-5) possibly due to estrogen production from aromatization of androgens (26).

The observation of a high incidence (67%) of gonadal tumors in CGD patients who presented with delayed puberty, but also had breast development, is in keeping with results from smaller studies (27). Germ cell tumors that produce androgens (28, 29) and estrogens (28, 30) may explain this presentation (31, 32). The finding highlights the importance of evaluating these patients for gonadal tumors. This study has included histological findings in a large number of individuals with PGD with the analysis undertaken in a single center of germ cell tumor expertise. This is the way forward for future prospective outcome studies to determine the precise risk for germ cell neoplasia in later life, particularly in PGD assigned male and retained gonads postpubertally.

A drawback of this study is its retrospective nature and based on a historical cohort of patients with a median age of 27 years. This possibly contributed to the variety in type of evaluation and patient management with inconsistent data reporting. Nevertheless, this study, to our knowledge, describes the phenotype and clinical course in the largest group of 46,XY GD patients by virtue of its international collaboration and access to the I-DSD registry.

## Data Availability

Some or all datasets generated during and/or analyzed during the current study are not publicly available but are available from the corresponding author on reasonable request.

## Abbreviations

AMH: anti-Mullerian hormone
CALIPER: Canadian Laboratory Initiative on Pediatric Reference Intervals
CGD: complete gonadal dysgenesis
DSD: differences of sex development
EGS: external genital score
GD: gonadal dysgenesis
hCG: human chorionic gonadotropin
PGDf: partial gonadal dysgenesis assigned female
PGDm: partial gonadal dysgenesis assigned male
UGT: undifferentiated gonadal tissue
